# The Network Structure of Decision-Making Competence in Chinese Adults

**DOI:** 10.3389/fpsyg.2020.563023

**Published:** 2020-09-18

**Authors:** Jiaxi Peng, Lei Ren, Nian Yang, Luming Zhao, Peng Fang, Yongcong Shao

**Affiliations:** ^1^ School of Psychology, Beijing Sport University, Beijing, China; ^2^ Department of Medical Psychology, Air Force Medical University, Xi’an, China; ^3^ Military Psychology Teaching and Research Section, Officers’ College of PAP, Chengdu, China; ^4^ HSBC Business School, Peking University, Shenzhen, China

**Keywords:** adult decision-making competence, network analysis, rational thought, strength, predictability

## Abstract

Decision-making competence refers to the ability to make better decisions, as defined by decision-making principles posited by models of rational choice. The adult decision-making competence (A-DMC) scale is a relatively mature evaluation tool used for decision-making competence. However, the A-DMC is yet far from other mature psychological evaluation tools, and especially the structure of A-DMC remains unclear. In the current study, we estimated a regularized partial correlation network of decision-making competence in a Chinese sample consisting of 339 adults who were evaluated by the A-DMC, and then the centrality indicators were calculated. The results revealed that all nodes of the decision-making competence networks are positively associated, except for the association of resistance to framing (RF) and resistance to sunk costs (SC). The strongest edge was between RF and applying decision rules (DR; regularized partial correlation = 0.37). The centrality indicators of RF and applying DR were highest, revealing that these two variables may play important roles in the decision-making competence network. Our study conceptualizes the decision-making competence from network perspectives, so as to provide some insights for future researches.

## Introduction

People make many decisions every day as they go about their lives. Some of these decisions are minor, but some are quite critical and shape our achievements and our lives, and the quality of these decisions related with the quality of life (Bruine de Bruin et al., 2020). Recently, several studies focused on the individual differences in decision-making quality. For example, why do some people routinely make poor decisions rooted in emotional reactions and impulsion? Why do some wise decision-makers and managers successfully overcome thinking inertia and heuristic bias under certain circumstances and make rational selections and judgments? Parker and Fischhoff (2005) developed the theory of decision-making competence to describe this individual difference in decision-making.

### Decision-Making Competence and Its Assessment

Decision-making competence refers to the ability to make better decisions, as defined by decision-making principles posited by models of rational choice (Bruine de Bruin et al., 2020). In other words, people with high decision-making competence generally make decisions in accordance with logical reasoning rather than emotion or intuition and seek the maximum utility option under given conditions (Shafir and LeBoeuf, 2002). Researchers have tried to measure decision-making competence in many ways (Lewis, 1981; Chiu et al., 2018). For example, some simulated decision-making games, such as the Iowa gambling task (IGT), balloon analogue risk task, and Cambridge gambling task (CGT), have been used to evaluate decision-making competence in many studies (Lauriola et al., 2013; Zois et al., 2014). Some studies have also used the decision-making scenarios developed by Lewis (1981) to evaluate decision-making ability, in which participants were asked to help a fictitious person to analyze and solve problems in three open ended dilemmas, and their responses were recorded and coded by whether participants mentioned other options, benefit, risk, long-term consequence, and advice seeking. It was deemed that the participants who mentioned these factors possessed greater decision-making competence. However, the degree to which these indicators accurately reflect decision-making competence is unclear. For instance, performance on the IGT may involve not only rationality but also working memory and consciousness of risk (Chiu et al., 2018).

In formal studies that attempted to measure decision-making, decision-making deficits or biases were examined. Tversky and Kahneman (1981) documented the existence of the framing effect and noted that decision-making and judgment can be changed by variation of the frames. Later, many studies found that the judgment and decision-making of humans often deviates from the principle of rationality, such as the sunk cost effect, negativity bias, omission bias, and the endowment effect (Tversky and Kahneman, 1981; West and Stanovich, 1997; Baron and Ritov, 2004; Scopelliti et al., 2015; Hood et al., 2016; Carstensen and DeLiema, 2018).

Biased decision-making seem to be stable across scenarios. For instance, West and Stanovich (1997) found that participants appearing overconfident in a motor skill game were significantly more overconfident in a knowledge assessment task. Baron and Ritov (2004) also found that with omission bias, people more easily accept losses due to their act of omission, rather than accepting the equivalent losses due to misconduct, and some participants exhibited consistent omission bias. Moreover, there is an internal consistency of these decision-making deficits, as it has been found that those who are more susceptible to the framing effect also tend to be more easily influenced by prior investment (sunk cost effect) or more often show omission bias and overconfidence (Stanovich and West, 2000). Scopelliti et al. (2015) documented that self-reported susceptibility to 14 decision-making biases were significantly positively correlated.

Additionally, decision-making biases were significantly correlated with both cognitive ability and cognition style (Thomas and Millar, 2012; Welsh et al., 2014). For instance, Welsh et al. (2014) found that education level and cognition style were moderately associated with the anchoring effect. Other studies implied that social science graduates who received systematic statistical training in schools will show less intuitive biases in decision-making tasks (Nisbett et al., 1983). Moreover, individuals with a higher education level behaved with less self-service biases (Toplak and Stanovich, 2003). Numeracy is positively correlated with the accuracy of risk perception (RP; Keller and Siegrist, 2009) and negatively correlated with the tendency to display overconfidence (Ghazal et al., 2014).

Based on the above data, decision-making biases were stable across scenarios, and correlated with each other, and were significantly correlated with both cognitive ability and cognition style. Then, Stanovich and other researchers believed that decision-making biases (e.g., the framing effect and the sunk cost effect) can reflect individual differences in rationality and decision-making competence to some extend (Stanovich and West, 2000; Parker and Fischhoff, 2005; Bruine de Bruin et al., 2007; Stanovich and Keith, 2015; Stanovich, 2018). However, a link is missing between the decision-making biases and decision-making competence. It is still unknown why and how these decision-making biases reflect decision-making competence.

### The Adult Decision-Making Competence Scale

Parker and Fischhoff (2005) believed that people required a suite of generally applicable decision-making skills when making a choice, and these skills are indeed the components of decision-making competence. They summarized four core decision-making skills: (1) assessing beliefs: whether the decision-maker can effectively perceive the probability of an event; (2) assessing values: whether the decision-maker can assess consequence options and be sensitive to option values but not to irrelevant information; (3) integration: combining beliefs and values coherently when making decisions. Better integration processes should result in selecting more appropriate decision rules (DR), and then executing them more accurately and consistently; and (4) metacognition: the decision-maker knows he has sufficient knowledge for the current task; he should not be blindly overconfident or underconfident or be hesitant due to lack of confidence, and should be able to clearly judge and recognize personal ability. Parker and Fischhoff (2005) suggested that people with higher core decision-making skills can make more optimal real-world decisions. Parker and Fischhoff (2005) suggested that these core decision-making skills can be reflected by decision-making bias or tasks. For example, consistency in RP and recognizing social norms (SN) are used to evaluate belief assessment; resistance to framing (RF) and resistance to SC are used to evaluate value assessment; applying DR and path independence are used to evaluate integration; and over- or under-confidence are used to evaluate meta-cognitive ability.

On this basis, Parker and Fischhoff (2005) developed the youth decision-making competence (Y-DMC) scale, which involves seven decision-making tasks and more than 100 items for assessing the decision-making competence of youth. Parker and Fischhoff (2005) found that the results of the seven tasks were significantly and positively correlated. The participants with lower Y-DMC scores reported more misbehaviors including drug abuse, dropping out of school, and violence during follow-up visits, while those with higher Y-DMC scores performed more optimally in real life, with less impulsion, higher education level, and participation in a complete family environment (Parker and Fischhoff, 2005).

On the basis of the Y-DMC, Bruine de Bruin et al. (2007) developed the adult decision-making competence (A-DMC) scale, which included six decision-making tasks (path independence was deleted because of the low test-retest reliability). Bruine de Bruin et al. (2007) found that individuals with lower A-DMC scores exhibited lower economic incomes, lower education level, increased abortion, excessive drinking, and drug abuse. Bavolar and Orosová (2015) reported that individuals with lower A-DMC scores experienced more psychological problems and decreased well-being. Parker et al. (2007) found that maximizing decision-making style was negatively correlated with decision-making competence. Carnevale et al. (2011) reported that the A-DMC score can predict the position levels of individuals in real society, and decision-making competence was significantly correlated with the need for cognition. In a follow-up study by Parker et al. (2018), the participants completed the Y-DMC and A-DMC at an interval of 11 years, and the results of the two scales were significantly correlated. Those with higher Y-DMC and A-DMC scale scores were less involved in crimes, marijuana smoking, or insecure behaviors. Peng et al. (2019) discovered that individuals with higher A-DMC scores performed better in the IGT and CGT. The A-DMC, because of its high reliability and validity, has been translated into Slovak, Chinese, and other languages and has been extensively used (Bavolar, 2013; Peng et al., 2019).

The core decision-making skills theory builds a bridge of decision-making biases and decision-making competence. Decision-making competence consists of the possession of four core decision-making skills, which can be reflected by six decision-making bias tasks (Bruine de Bruin et al., 2020).

### Network Analysis

Network analysis is a rapidly emerging analytical method that can describe the relationships among psychological variables. In network analysis, the observed variables are regarded as nodes, and the between-node edges are regarded as the correlations between observational variables. Under the drive of data, a network composed of nodes and edges can assist with visualizing the relationships among variables (Borsboom, 2008). In psychology research, due to the presence of numerous nodes, false correlations easily exist: if two nodes are both correlated with a third node, even if these two nodes are not directly related, they may still be statistically analyzed as a significant correlation (Pourahmadi, 2011). To avoid any misguidance, Pourahmadi (2011) proposed a solution and used the partial correlation coefficient in network analysis to more accurately reflect the real between-node associations. Partial correlation networks, which have also been called Gaussian graphical models (GGMs), can offer routes to accurately explore between-node relationships (Borsboom and Cramer, 2013). In addition, network analysis allows assessment of the importance of each variable in the network by providing some centrality indices. For centrality analysis, the commonly used indices include strength, closeness, and betweenness. Recent research shows that there is a greater amount of stability and reliability with strength centrality as compared to closeness and betweenness centrality, and closeness and betweenness centrality seem especially unsuitable as assessments of node importance in psychological networks (Epskamp et al., 2017; Bringmann et al., 2019).

### The Aim of the Current Study

Although the A-DMC is a relatively mature tool for evaluating decision-making competence, many problems remain to be solved before it can be considered as reliable as other mature psychological evaluation tools, such as the Wechsler intelligence scale. For example, the structure of the A-DMC remains somewhat unclear. The basic hypothesis of mainstream latent variable models is that there is a latent variable such as decision-making competence that is represented by its indicators, namely observed variables such as RF and DR. The correlations between observed variables are fully explained by the shared latent variable, which indicates that there is a local independence in the observed variables, and the relationship between these observed variables is not the focus of the models (Bollen, 2002). However, whether there is a latent general factor in the decision-making competence remains controversial. For instance, Stanovich (2018) noted that the internal consistency coefficient of a series of decision tasks was far lower than that of traditional psychological evaluation tools, with a possible explanation being that rational thought may have several mutually independent dimensions or factors, and these dimensions are largely different. However, all dimensions were closely correlated with the results of decision-making, and all were indispensable to rational thought skills. Although previous studies tried to examine the factor structure of the A-DMC, they have rarely expected or proposed the existence of a general factor. In an initial exploratory factor analysis of A-DMC, Bruine de Bruin et al. (2007) suggested that the two-factor model of A-DMC was also acceptable. Peng et al. (2019) analyzed the confirmatory factors of A-DMC and found that the factor loadings of the six dimensions greatly vary, from 0.18 to 0.77, suggesting that the significance of each dimension may be different. Additionally, Parker and Fischhoff (2005) hold that RF and resistance to SC are used for value assessment, but many studies show that the scores of these two dimensions are not significantly correlated (Bruine de Bruin et al., 2007; Peng et al., 2019).

A network analysis does not necessarily depend on the definitions of latent variables, and it is not based on the hypothesis that the observed variables are related due to their shared common latent variable. Instead, network analysis is purely based on the modeling of the observed variables. Hence, the relationships between observed variables can be directly analyzed (Clifton and Webster, 2017). At present, no research has been performed on the network analysis of decision-making competence, but network analysis will offer some new perspectives for studies examining decision-making competence. First, network analysis does not need to hypothesize that there is a G factor in the six decision-making bias tasks for A-DMC, but instead, on a data-driven basis, it shows how the six decision-making task results are correlated with each other without the strict limit of latent variable models. Second, of the six decision-making bias tasks, network analysis can reveal the ones that are at the core of decision-making competence assessment. Additionally, although the results of previous studies indicated that there were significant correlations among the subscales of A-DMC, there is doubt whether these correlations were real. For example, A and B may be correlated because they are both related with C, but after controlling for the effect of C, A and B may be not significantly correlated with each other. In the network analysis, all the correlations were regularized partial correlations, which are more convincing.

## Materials and Methods

### Participants

Using a convenient sampling method, a total of 384 participants were recruited from five communities. To avoid the homogeneousness of participants, the five chosen communities consisted with a university affiliated community, where residents were all university teachers or their families, and most of them were well educated; a villager resettlement community, in which many residents were less educated; and three common urban communities. All participants were informed regarding the objectives and implications of this research, and all signed informed consent forms before the A-DMC tests. Because of the large number of items in the A-DMC, it is likely that many participants will skip some items. To avoid this problem, we presented the items of the A-DMC on a notebook computer and displayed one item on the screen at a time so that the participant was required to finish an item before proceeding ahead to the next item. Among the 384 participants, 42 participants abandoned the test and did not complete all the items, and three participants answered the items following obvious rules (e.g., selection of answer A for all items). The remaining 339 participants consisted of 191 males and 148 females who were aged 18–51 years old, with a mean of 31.22 years (*SD* = 5.12). Their education levels included middle school and below (*n* = 37), high school (56), university/college (179), and 67 (postgraduate and above). The testers in charge were 12 trained postgraduates majoring in psychology. They mainly introduced the research objectives to the participants and informed the participants that the answers were not marked right or wrong. The data were collected from the activity rooms of the communities. Ten notebook computers were provided to the participants and were separated at a distance greater than 5 m to avoid mutual interference. The participants who finished all items were awarded 150 Yuan (approximately 24 U.S. dollars).

### A-DMC Scale

The A-DMC developed by Bruine de Bruin et al. (2007) consisted of six scales. Peng et al. (2019) introduced the A-DMC into China, and subsequently localized and revised it. The Chinese version of the A-DMC has demonstrated high reliability and validity. The six subscales of the A-DMC are outlined below.

**Resistance to framing (RF)** evaluates whether an individual is influenced on how information is presented, and specifically compares between negative framing and positive framing of the information. This subscale includes seven risky choice framing tasks and seven attribute framing tasks described in positive and negative frames, respectively. Some example items are “An unusual disease is expected to kill 600 people. If option A is adopted, 200 people will be saved (400 people will die); If option B is adopted, there is a 1/3 chance 600 people will be saved (no people will die), and a 2/3 chance no people will be saved (all people will die). Which option do you prefer?” (i.e., risky choice framing task) and “80% lean (20% fat) ground beef. What’s your evaluation of the quality of this ground beef?” (i.e., attribute framing task). Responses were assigned on a six-point scale ranging from 1 = definitely would choose A/very low to 6 = definitely would choose B/very high. If a participant showed greater consistency when making decisions when facing the same problems in positive and negative frames, then he or she was less susceptible to the framing effect. The results revealed a mean absolute difference of 5 between related frames.

**Recognizing social norms (SN)** consists of descriptions of 16 negative events. Participants were first asked to state whether they accepted a negative event (e.g., “Do you think it’s sometimes OK to steal under certain circumstances?” Yes/No). Then, they were asked to evaluate what proportion of the public might accept this event (e.g., “Out of 100 people, how many would say it is sometimes OK to steal under certain circumstances?” 0–100%). The actual proportion was determined after data collection. The overall score is represented as a rank correlation between the judged proportion and actual proportion, with a higher correlation coefficient indicating more accurate perceptions of SNs.

**Under/overconfidence (UOC)** consists of 34 items. Participants were first asked to state whether or not items were correct (e.g., “There is no way to improve your memory” True/False) and then evaluate their confidence in their answer from 50% (just guessing) to 100% (absolutely sure). The overall score was characterized by one minus the absolute difference between mean confidence and percent correct on all items, with a higher score indicating more accurate self-cognition.

**Consistency in risk perception (RP)** consists of 20 items. Participants were asked to evaluate the probability of an event occurring (e.g., relocating to another province and dying from a traffic accident). The time frame was within 1 year on 10 items and within 5 years on another 10 items. Response options ranged from 0% (no chance) to 100% (certainty). Scores on this subscale were counted using a paired method: the 20 items were mutually paired to form 20 logical relationships. For instance, the probability of migrating to another province within 1 year should be smaller than that within 5 years; dying from a terrorist attack was a subset of death by accident, as the probability of the former occurring is smaller. The score of this subscale is the percentage of consistent risk judgment pairs. A higher score on this subscale suggests the individual is more rational in estimating the probability of an event.

**Applying decision rules (DR)** assesses the ability of participants to solve practical problems according to predetermined decision-making rules. This subscale consists of 15 items. Participants choose one of five TV sets according to the limited conditions provided in the items (e.g., picture quality and brand). The overall score is the percentage of correct answers.

**Resistance to sunk costs (SC)** measures the ability to ignore prior investments when making current decisions. This subscale involves 10 items, such as “After a large meal at a restaurant, you order a big dessert with chocolate and ice cream. After a few bites, you find you are full and you would rather not eat any more of it. Would you be more likely to eat more or to stop eating it?” Responses spanned a six-point scale, ranging from 1 = most likely to eat more to 6 = most likely to stop eating. A larger score indicates the participant can more easily ignore a previous investment and make a more rational decision.

### Network Analysis

The decision-making competence networks were computed *via* a GGM running the R package qgraph (Epskamp et al., 2012, 2018b). GGMs are undirected networks in which the edges represent partial correlations between two nodes after controlling for all other nodes in the network (Epskamp and Fried, 2018). In the present study, the GGMs were calculated basing on nonparametric Spearman rho correlation matrices, and we provided the nonparametric Spearman rho correlations among the variables in Table 1. All edges were regularized (edges with small partial correlations were regularized to zero) *via* the graphical least absolute shrinkage and selection operator (LASSO) algorithm to obtain a sparse network with higher stability and interpretability (Friedman et al., 2008; Epskamp and Fried, 2018). As recommended in a previous study, we set the GGM tuning parameter to a value of 0.5 to accurately judge and weigh the sensitivity and specificity of discerning true edges (Drton et al., 2011). In the final visualized networks, the thicknesses of edges represent the degrees of the partial correlations. Blue edges represent positive partial correlations, whereas red edges represent negative partial correlations.

**Table 1:** Descriptive statistics, network analysis results, and nonparametric Spearman rho correlation matrix of adult decision-making competence (A-DMC) subscales.

	Mean	*SD*	Strength	Predictability	DR	RF	RP	SN	SC
DR	0.62	0.21	0.88	0.40					
RF	4.00	0.60	1.04	0.37	0.50^**^				
RP	0.69	0.09	−1.31	0.19	0.29^**^	0.36^**^			
SN	0.50	0.21	0.21	0.31	0.29^**^	0.36^**^	0.26^**^		
SC	4.19	0.87	0.31	0.28	0.41^**^	0.27^**^	0.33^**^	0.40^**^	
UOC	0.77	0.08	−1.13	0.18	0.31^**^	0.30^**^	0.26^**^	0.38^**^	0.27^**^

** *p* < 0.01.

DR, applying decision rules; RF, resistance to framing; RP, consistency in risk perception; SN, recognizing social norms; SC, resistance to sunk costs; UOC, under/overconfidence.

The relative importance of each node in the decision-making competence network was examined using the node strengths in the R package qgraph (Epskamp et al., 2012). Node strength is defined as the sum of the absolute value of edge weights attached to a node. Higher node strength values represent greater importance in the network. In addition, we calculated the predictability of each node by running the R package mgm (Haslbeck and Fried, 2017). Node predictability is quantified as the percentage of shared variance of each node with all its neighboring nodes, which represents the upper bound of a given node’s controllability (Haslbeck and Fried, 2017). Finally, we computed the small-worldness value for the present network by using the R package qgraph (Epskamp et al., 2012). This value represents the transitivity of networks. In a small-world network, each node can quickly affect other nodes in the network. A small-worldness value higher than three is considered to possess the small-world property (Humphries and Gurney, 2008).

The robustness of the present network was examined *via* running the R package bootnet (Epskamp et al., 2018a). First, we examined the accuracy of edge weights by computing 95% confidence intervals (CI) for each edge using a nonparametric bootstrap approach with 2,000 bootstrap samples. Second, we examined the stability of node strengths by calculating the correlation stability (CS) coefficient running a case-dropping bootstrap approach. The value of the CS-coefficient should not be less than 0.25, and preferably higher than 0.5 (Epskamp et al., 2018a). Third, we conducted bootstrapped difference tests (*α* = 0.05) for edge weights and node strengths, with 2,000 bootstrap samples, to examine whether there are significant differences between two edge weights or two node strengths.

## Results

The descriptive statistics for each subscale of the A-DMC and the nonparametric Spearman rho correlation matrix of each subscale of the A-DMC is shown in Table 1.

**Figure 1:**
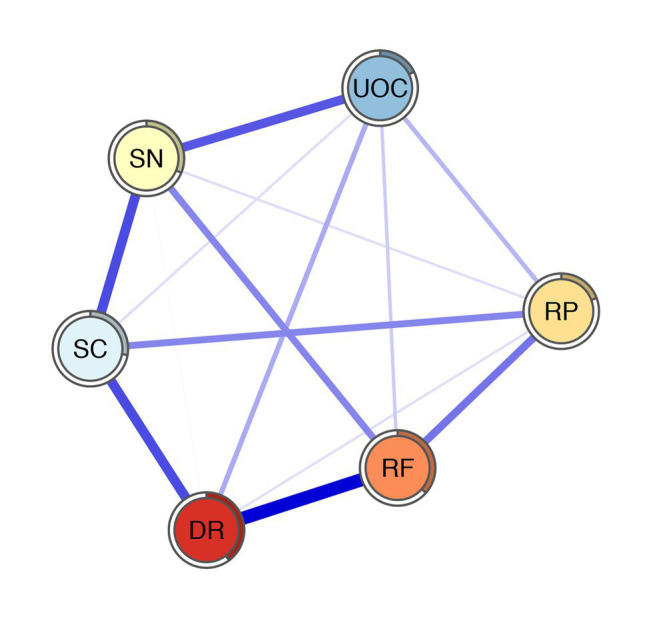
The network of A-DMC. DR, applying decision rules; RF, resistance to framing; RP, consistency in risk perception; SN, recognizing social norms; SC, resistance to sunk costs; UOC, under/overconfidence. The thicknesses of edges represent the degrees of correlations. Blue edges represent positive correlation and red edges represent negative correlation. The rings around nodes depict its predictability.

The decision-making competence network is shown in Figure 1. Several characteristics of the network can be observed. First, out of 15 possible edges, 14 (93.3%) are non-zero, of which all are positive, indicating that there are general associations between nodes except the association between SC and RF. Second, there are several strong associations between RF and DR (regularized partial correlation = 0.37), SC and SN (regularized partial correlation = 0.26), DR and SC (regularized partial correlation = 0.26), and SN and UOC (regularized partial correlation = 0.24). Third, the node predictability ranges from 0.18 to 0.40, with an average of 0.29 (see Table 1). UOC has the lowest predictability: 18% of its variance can be explained by its neighboring nodes. DR has the highest predictability: 40% of its variance can be explained by its neighboring nodes. RF has the second highest predictability: 37% of its variance can be explained by its neighboring nodes.

**Figure 2:**
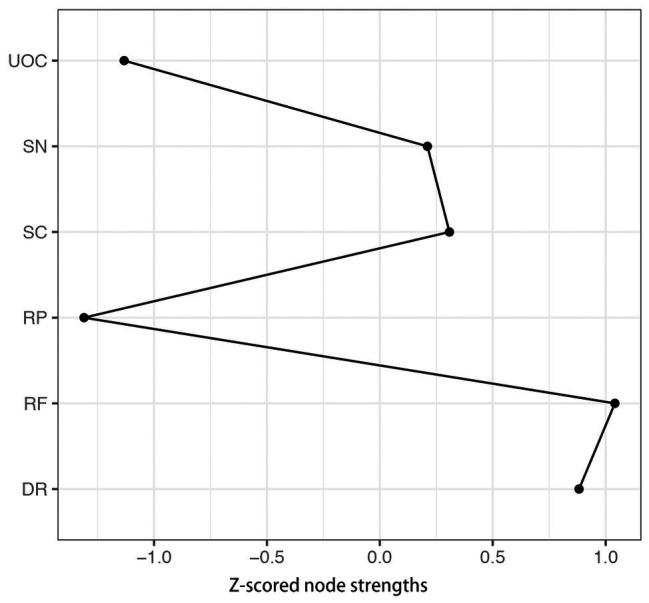
The *z*-scored value of strength for each node in the A-DMC network. DR, applying decision rules; RF, resistance to framing; RP, consistency in risk perception; SN, recognizing social norms; SC, resistance to sunk costs; UOC, under/overconfidence.

The *z*-score value of strength for each node in the network is shown in Figure 2. RP has the lowest strength value. RF exhibits the highest strength value. This indicates that RF is the most connected node in the present network from a statistical point of view. DR has the second highest strength value. The small-worldness value of the present network is 1.00, indicating that it does not have the small-world property.

**Figure 3:**
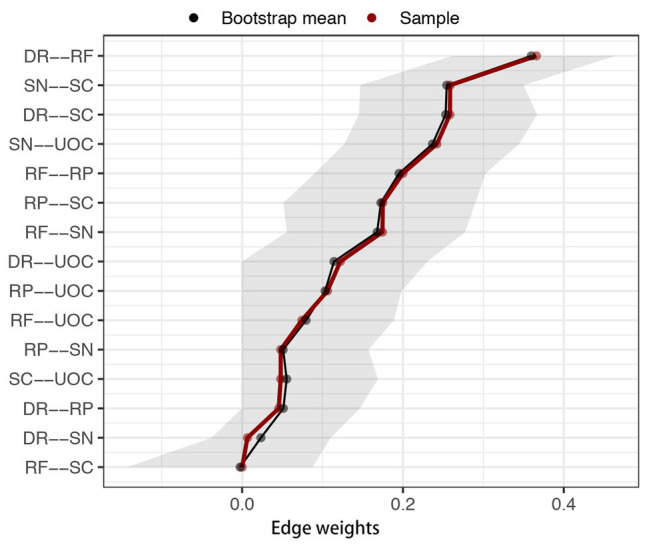
Bootstrapped 95% confidence intervals (CI) of edge weights. DR, applying decision rules; RF, resistance to framing; RP, consistency in risk perception; SN, recognizing social norms; SC, resistance to sunk costs; UOC, under/overconfidence. The red line indicates the sample edge weight (sorted in increasing order in *x*-axis) and the gray bar is the bootstrapped CI.

**Figure 4:**
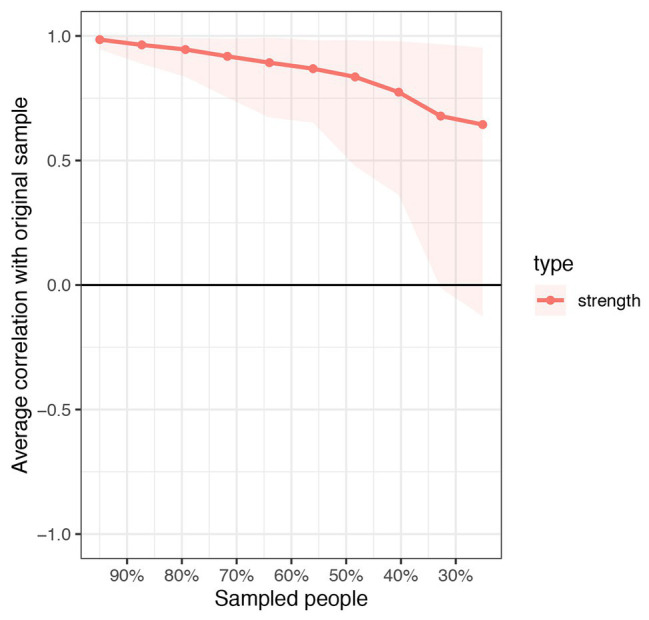
Stability of node strengths. The red bar represents the average correlation between strength in the full sample and subsample with the red area depicting the 2.5th quantile to the 97.5th quantile.

**Figure 5:**
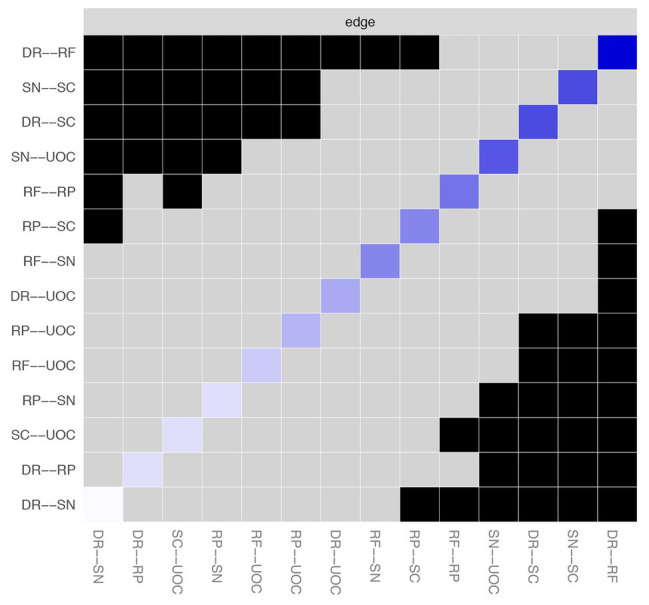
Bootstrapped difference test for edge weights. DR, applying decision rules; RF, resistance to framing; RP, consistency in risk perception; SN, recognizing social norms; SC, resistance to sunk costs; UOC, under/overconfidence. Gray boxes indicate edge weights that do not differ significantly from one another, while black boxes indicate edge weights that do differ significantly. Blue and red boxes on the diagonal correspond to edge weights with positive and negative correlations, respectively.

**Figure 6:**
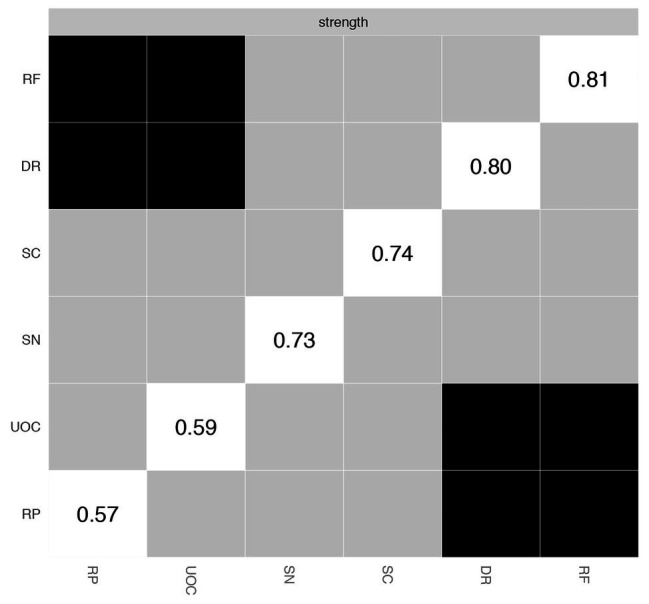
Bootstrapped difference test for node strengths. DR, applying decision rules; RF, resistance to framing; RP, consistency in risk perception; SN, recognizing social norms; SC, resistance to sunk costs; UOC, under/overconfidence. Gray boxes indicate node strengths that do not differ significantly from one another, while black boxes indicate node strengths that do differ significantly. The number in the white boxes (i.e., diagonal line) represents the value of node strengths.

Figure 3 depicts the accuracy of edge weights. In consideration of the present network, which is derived from 339 individuals and only has six nodes, the estimation of edge weights is accurate. Figure 4 depicts the stability of node strengths. The CS coefficient of node strength is 0.44, which indicated that the estimation of node strengths is stable. Figure 5 depicts the bootstrapped difference test for edge weights, and Figure 6 depicts the bootstrapped difference test for node strengths. These results indicate that a small to moderate proportion of the differences among edge weights and node strengths is statistically significant in the present network.

## Discussion

In this study, network analysis was applied to the A-DMC. This is the first time that network analysis has been applied to the evaluation of decision-making competence. Network theory provides an alternative method to conceptualize psychological constructs, which regards psychological constructs as interacting systems, and their components interact with each other, and actively participate in the emergence of this construct rather than the passive indicators of this construct (Briganti et al., 2019). Thus, it is reasonable to see decision-making competence as an interacting system, consisting of different decision-making bias tasks based on this theory, which provides a new perspective to describe and understand decision-making competence. Network analysis is a related analytical tool that can be used to try to disclose the structure of psychological constructs. It is able to mathematically analyze and visually display the relationships (usually refers to the regularized partial correlations) among complex variables and is not dependent on prior assumptions of causality among variables (Dalege et al., 2017). Particularly, as compared to mere correlational approaches, network analysis can be used to examine the extent to which variables are central to the network.

An increasing number of studies have used network analysis to provide a concept map for related psychological constructs, including attitudes (Dalege et al., 2017), stigma (Zihan et al., 2020), self-worth (Briganti et al., 2019), empathy (Briganti et al., 2018), and personality (Costantini et al., 2015). The primary goal is to explore relationships among components of decision-making competence. Secondly, centrality indices are used to quantify the importance of each component in the present network, so as to provide some insights for future research and related training. As (Bruine de Bruin et al., 2012) suggested “If critical decision-making skills can be improved through teaching and decision support, the research on decision-making competence ultimately brings the promise of designing interventions that improve the outcomes of people’s decisions.” Network analysis could help us to find which decision-making skill is the most important in the decision-making competence network.

Our results showed that all nodes of decision-making competence networks are positively associated (except that regularized partial correlation was not found between RF and SC). Although significant positive correlations between subscales of the A-DMC have also been reported in previous studies (Bruine de Bruin et al., 2007; Peng et al., 2019), they were Pearson correlations or rank correlations. In this study, a partial correlation with regularization was found, and the false correlation induced by a covariation relationship with the other variables was excluded. Hence, partial correlation with regularization was used in network analysis to more accurately characterize the real relationships between nodes.

The results of edges showed that DR and RF were the most closely related. Close associations were also found between DR and SC, between SC and SN, and between SN and UOC, which is consistent with previous studies (Bruine de Bruin et al., 2007). No regularized partial correlation was found between RF and SC, which is consistent with the results of Bruine de Bruin et al. (2007) and Peng et al. (2019). The possible reason for this may be that RF and SC may not be adequate in jointly evaluating the value assessment as hypothesized by Parker and Fischhoff (2005) or that although RF and SC partially reflected the value assessment, they separately assessed other psychological traits. For instance, SC uncovered the bravery and decisiveness of individuals in abandoning previous wrong investments (Soman, 2001), but RF reflected the view regarding justice and emotional heuristic processing (Mai et al., 2018; Yao et al., 2018).

The predictability of different nodes was largely different and varied from 40% (DR) to 18% (UOC), with a mean of 29%, indicating that the average node variance of 29% was explained by all its neighboring nodes in the networks. In particular, it should be noted that predictability is the upper bound estimation (Haslbeck and Fried, 2017). UOC and RP were the most unpredictable and the most predictable of all nodes, respectively. The centrality results showed that RF and DR had the highest values of strength centrality in the decision-making competence network, indicating that these two nodes are most strongly associated with other nodes in the network. It is indicated that RF and DR are located at very core positions in the decision-making competence network (Marcus et al., 2018). The results suggest that during the evaluation of decision-making competence, the results in RF and DR may be highly important. To train or promote decision-making competence, we should also note whether the decision-makers complied with decision-making rules or ignored the information presentations. This is because targeting nodes with higher centrality could lead to general benefits in the rest of the other nodes considered in the network.

Based on the results of our study, we can conclude that the network connectivity of decision-making competence was high, and close associations were found among nodes. The values of strength centrality of RF and DR were the highest, and the two notes may play important roles in the decision-making competence network. This study has some limitations. First, network analysis cannot define a psychological trait or concept. The system or network composed by observed variables is not the conception of the variable; in other words, the decision-making competence network is not decision-making competence itself. Second, network analysis is only a method to describe the connections of the observed variables, and the results are sample-specific. Although some studies have documented the high repeatability of network analysis results (Borsboom, 2017), caution should be applied as to whether our findings can be applied to other populations. Additionally, a convenient sampling method was used in the current study. Although we tried to avoid subject consistency and enrolled participants from different communities, there is actually no clear targeted population. Thirdly, because we used cross-sectional data to carry out the analyses, we cannot determine the direction of edges. For instance, we cannot interpret whether the most central item activates other items, is activated by other items, or both. Fourthly, the A-DMC focuses on only selected six competencies, and the current analysis provides a picture of only them. Numerous other competencies can be identified with the potential to affect the found network. Finally, we found again that RF and SC were not significantly associated, which is contrary to theories of core decision-making skills, but we did not fully explain this phenomenon, and therefore, further exploration in the future is warranted.

## Data Availability Statement

The raw data supporting the conclusions of this article will be made available by the authors, without undue reservation.

## Ethics Statement

The studies involving human participants were reviewed and approved by the Ethics Committee of the Air Force Medical University. The patients/participants provided their written informed consent to participate in this study.

## Author Contributions

JP, PF, LR, and YS conceived and designed the study. JP and LZ collected the data. JP and LR analyzed the data. LR, NY, and YS contributed reagents, materials, and analysis tools. JP, PF, LR, and YS wrote the manuscript. All authors contributed to the article and approved the submitted version.

### Conflict of Interest

The authors declare that the research was conducted in the absence of any commercial or financial relationships that could be construed as a potential conflict of interest.
